# High Rates of Antimicrobial Drug Resistance Gene Acquisition after International Travel, the Netherlands

**DOI:** 10.3201/eid2004.131718

**Published:** 2014-04

**Authors:** Christian J.H. von Wintersdorff, John Penders, Ellen E. Stobberingh, Astrid M.L. Oude Lashof, Christian J.P.A. Hoebe, Paul H.M. Savelkoul, Petra F.G. Wolffs

**Affiliations:** Maastricht University Medical Center, Maastricht, the Netherlands (C.J.H. von Wintersdorff, J. Penders, E.E. Stobberingh, A.M.L. Oude Lashof, C.J.P.A. Hoebe, P. Savelkoul, P.H.M. Wolffs);; South Limburg Public Health Service, Geleen, the Netherlands (C.J.P.A. Hoebe)

**Keywords:** ESBL, CTX-M, quinolone, resistance genes, qnrB, qnrS, intestinal microbiota, metagenomic, traveling, antibiotics, antimicrobial, antibacterial, the Netherlands, Shewanella algae, Kluyvera, Enterobacteriaceae, bacteria

## Abstract

We investigated the effect of international travel on the gut resistome of 122 healthy travelers from the Netherlands by using a targeted metagenomic approach. Our results confirm high acquisition rates of the extended-spectrum β-lactamase encoding gene *bla*_CTX-M_, documenting a rise in prevalence from 9.0% before travel to 33.6% after travel (p<0.001). The prevalence of quinolone resistance encoding genes *qnrB* and *qnrS* increased from 6.6% and 8.2% before travel to 36.9% and 55.7% after travel, respectively (both p<0.001). Travel to Southeast Asia and the Indian subcontinent was associated with the highest acquisition rates of *qnrS* and both *bla*_CTX-M_ and *qnrS*, respectively. Investigation of the associations between the acquisitions of the *bla*_CTX-M_ and *qnr* genes showed that acquisition of a *bla*_CTX-M_ gene was not associated with that of a *qnrB* (p = 0.305) or *qnrS* (p = 0.080) gene. These findings support the increasing evidence that travelers contribute to the spread of antimicrobial drug resistance.

Antimicrobial drug resistance is a public health threat worldwide that limits clinical treatment options for bacterial infections. Most research on antimicrobial drug resistance has been focused on resistance in clinically relevant pathogenic bacteria. However, a vast and largely unexplored reservoir of resistance genes is present in nonpathogenic bacteria living in the environment or as commensal agents (*1*–*5*). Because of horizontal gene transfer (HGT) among microbes of diverse species and genera, antimicrobial drug resistance mechanisms in an organism, regardless of whether it is a pathogen, have the potential to emerge in clinically relevant pathogens (*6*). Several of such HGT interactions between clinically relevant pathogens and environmental species have been described; for example, the plasmid-mediated quinolone resistance encoding *qnrA* gene originated from the chromosomes of the aquatic bacterium *Shewanella algae* (*7*). Another well-known example is the extended-spectrum β-lactamase (ESBL) encoding *bla*_CTX-M_ gene, which originates from chromosomal genes of environmental *Kluyvera* species (*8*) and has emerged as the most prevalent cause of plasmid-mediated ESBL.

Resistance reservoirs have unpredictable and immense potential for rendering antimicrobial drugs ineffective. The human gut microbiota warrants special attention because of its high density of microorganisms and high accessibility (*9*). The gastrointestinal tract is constantly exposed to numerous bacteria from the environment, e.g., food, water, soil, other humans, or animals. These incoming bacteria often harbor antimicrobial drug resistance genes (*10*), which can be transferred to the indigenous microbial communities through HGT, where they may enrich the pool of available antimicrobial resistance elements in the gut microbiota.

Potential for intercontinental transfer of antimicrobial drug–resistant bacteria in the microbiota necessitates studies that focus on the antimicrobial resistance of the gut microbiome as a whole, the so-called “gut resistome,” by using culture-independent metagenomic approaches (*9*). Metagenomic approaches avoid the bias that is introduced when selective culturing is applied because ≈80% of the gut microbiota is not cultivatable (*11*). 

Travel to geographic areas in which rates of bacteria that are resistant to antimicrobial drugs are high has been indicated as a risk factor for the acquisition of such bacteria (*12*). Studies in Australia (*13*), Sweden (*14*,*15*), and the Netherlands (*16*) have shown that international travel is a major risk factor for colonization with ESBL-producing *Enterobacteriaceae*. Likely, these resistant strains are acquired from the environment during travel, e.g., through food consumption (*17*). Because the human intestinal microbiome will come in contact with many different bacterial species from travel-related environments, the effect of international travel on antimicrobial drug resistance is most likely limited to neither opportunistic pathogens, such as *Escherichia coli*, nor to ESBL-encoding resistance genes.

In this study, we aimed to investigate the effect of international travel on the human gut resistome. By using a targeted (PCR-based) metagenomic approach, we compared the presence and relative abundance of specific resistance determinants in the entire human gut microbiome before and after international travel.

## Materials and Methods

### Population and Design

Healthy long-distance travelers were recruited during November 2010–August 2012 through travel clinics (EASE Travel Clinic & Health Support, www.ease-travelclinic.nl/en/) located in the southern part of the Netherlands. Travelers consenting to participate were asked to collect a fecal sample before and immediately after travel and to provide records of the duration and destination of their travel, illnesses or complaints during travel, drug use, and antimicrobial drug use within the 3 months preceding travel. The fecal samples were sent to clinics by regular mail on the same day of collection and were processed on the day of receipt. The study comprised 122 travelers.

The countries visited were categorized into geographic regions. These regions were Southeast Asia (Asia excluding the Indian subcontinent and the Middle East), the Indian subcontinent (Bangladesh, Bhutan, India, Nepal, Pakistan, and Sri Lanka), northern Africa (countries north of the equator), southern Africa (countries south of the equator), southern Europe, Central America, and South America.

### Fecal Specimen Processing and DNA Extraction

Fecal samples were diluted 10-fold in peptone/water solution (Oxoid, Basingstoke, UK) containing 20% (vol/vol) glycerol (Merck, Darmstadt, Germany) and homogenized by vortexing. They were stored at −20°C until molecular analysis was performed.

For the extraction of metagenomic DNA, 200 μL of diluted feces was added to a 2-mL vial containing 0.5 g of 0.1 mm zirconia/silica beads (BioSpec, Bartlesville, OK, USA), 4 glass beads, 3.0–3.5 mm (BioSpec), and 1.2 mL of lysis buffer from the PSP Spin Stool Kit (Stratec Molecular, Berlin, Germany). Samples were disrupted in a Magna Lyser device (Roche, Basel, Switzerland) in 3 cycles of 1 min. at 5,500 rpm. Subsequently, metagenomic DNA was isolated from the samples by using the PSP Spin Stool Kit according to the manufacturer’s instructions. DNA was eluted in 200 μL elution buffer and stored at −20°C until further analysis.

### Real-time PCR

Real-time PCR was performed to detect and quantify the β-lactamase–encoding genes *cfxA*, *bla*_CTX-M_, and *bla*_NDM_; tetracycline resistance­–encoding genes *tetM* and *tetQ*; macrolide resistance–encoding gene *ermB*; aminoglycoside resistance­–encoding gene *aac(6*′*)-aph(2′′)*; and quinolone resistance encoding genes *qnrA*, *qnrB*, and *qnrS*. The 16S rDNA was amplified as a reference gene to normalize for the amount of bacterial DNA in the samples.

The 16S rDNA, *cfxA*, *tetM*, *tetQ*, *ermB*, and *aac(6′)-aph(2′′)* targets were amplified by using a MyiQ Single-Color Real-Time PCR Detection System (BioRad, Hercules, CA, USA) in 25-μL reactions containing 12.5 μL iQ SYBR Green Supermix (BioRad) and 5-μL template DNA. Melting curves were checked for each sample to confirm amplification of the correct product. For every target, amplified PCR products of 10 random positive samples were separated by agarose gel electrophoresis to control for purity and size of the amplicons. Finally, for all genes except the 16S rDNA (because of expected heterozygous amplicons), these products were sequenced by using the PCR primers and an ABI BigDye Terminator v1.1 Cycle Sequencing Kit (Applied Biosystems, Foster City, CA, USA). Sequencing data were obtained by using an ABI 3730 DNA Analyzer (Applied Biosystems) and were analyzed by using BLAST (http://blast.ncbi.nlm.nih.gov/Blast.cgi).

The *bla*_CTX-M_, *bla*_NDM_, *qnrA*, *qnrB*, and *qnrS* genes were amplified on a 7900HT Fast Real-Time PCR System (Applied Biosystems) in 25-μL reactions containing 12.5 μL ABsolute QPCR ROX Mix (Thermo Scientific, Waltham, MA, USA) and 10-μL template DNA. The *bla*_CTX-M_ assay enables identification of the various phylogenetic groups by use of 4 probes. The probes to detect *bla*_CTX-M_ groups 1 and 2 were combined in the first reaction, and the probe to detect *bla*_CTX-M_ group 9 was combined with a probe to detect all groups except for the CTX-M-1 group in a second reaction. All primer and probe sequences and PCR conditions for each target are displayed in Table 1.

**Table 1:** PCR primer/probe sequences and additional PCR conditions to identify antimicrobial resistance genes in gut microbiota after international travel, the Netherlands, 2010–2012

Primer/probe	Sequence,* 5′→3′	Final conc., nM	Amplicon size, bp	Cycling conditions	Ref.
16S-rDNA_F	TGGAGAGTTTGATCCTGGCTCAG	500	526	95°C, 4 min	(*18*)
16S-rDNA_R	TACCGCGGCTGCTGGCAC	250		35 × 95°C, 15 s; 65°C, 60 s	
cfxA_F	TGACAGTGAGAGATTTGCTGC	300	150	95°C, 3 min	(*19*)
cfxA_R	GGTCAGCCGACATTTCCTCTT	300		40 × 95°C, 15s; 60°C, 15s; 72°C, 30s	
tetM_F	ACACGCCAGGACATATGGAT	300	126	95°C, 3 min	(*19*)
tetM_R	GGGAATCCCCATTTTCCTAA	300		40 × 95°C, 15s; 57°C, 15s; 72°C, 30s	
tetQ_F	CAAGGTGATATCCGCTCTGA	300	128	95°C, 3 min	(*19*)
tetQ_R	GGAAAATCGTTCTTCCAGCA	300		40 × 95°C, 15s; 57°C, 15s; 72°C, 30s	
ermB_F	AAGGGCATTTAACGACGAAACTG	300	438	95°C, 3 min	This study
ermB_R	ATTTATCTGGAACATCTGTGGTATG	300		40 × 95°C, 20s; 60°C, 30s; 72°C, 40s	
aac6-aph2_F	TTGGGAAGATGAAGTTTTTAGA	300	173	95°C, 3 min	(*20*)
aac6-aph2_R	CCTTTACTCCAATAATTTGGCT	300		40 × 95°C, 15s; 57°C, 20s; 72°C, 30s	
CTX-M_F	ATGTGCAGYACCAGTAARGTKATGGC	500	336	95°C, 15 min	(*21*)
CTX-M_R	ATCACKCGGRTCGCCNGGRAT	500		40 × 95°C, 15s; 58°C, 20s	
CTX-M-1	JOE-CCCGACAGCTGGGAGACGAAACGT-BHQ1	100		72°C, 30s	
CTX-M-2	6FAM-CAGGTGCTTATCGCTCTCGCTCTGTT-BHQ1	100			
CTX-M-9	JOE-CTGGATCGCACTGAACCTACGCTGA-BHQ1	100			
CTX-M-2-8-9-25	6FAM-CGACAATACYGCCATGAA-MGB-NFQ	100			
NDM_F	ATTAGCCGCTGCATTGAT	400	154	95°C, 15 min	(*22*)
NDM_R	CATGTCGAGATAGGAAGTG	400		42 × 95°C, 15s; 60°C, 60s	
NDM_probe	6FAM- CTG[+C]CA[+G]AC[+A]TT[+C]GGTGC-BHQ1	200			
qnrA_F	CAGTTTCGAGGATTGCAGTT	400	148	95°C, 15 min	(*23*)
qnrA_R	CCTGAACTCTATGCCAAAGC	400		45 × 95°C, 30s; 52°C, 30s;	
qnrA_probe	6FAM-AAGGGTGYCACTTCAGCTATGCC-BHQ1	100		72°C, 30s	
qnrB_F	CAGATTTYCGCGGCGCAAG	400	134	95°C, 15 min	(*23*)
qnrB_R	TTCCCACAGCTCRCAYTTTTC	400		45 × 95°C, 30s; 55°C, 30s;	
qnrB_probe	6FAM-CGCACCTGGTTTTGYAGYGCMTATATCAC-BHQ1	100		72°C, 30s	
qnrS_F	TCAAGTGAGTAATCGTATGTA	400	157	95°C, 15 min	(*23*)
qnrS_R	GTCTGACTCTTTCAGTGAT	400		45 × 95°C, 30s; 55°C, 30s	
qnrS_probe	6FAM-CCAGCGATTTTCAAACAACTCAC-BHQ1	100		72°C, 30s	

*Nucleic acids between brackets and preceded by + are locked nucleic acids; nM, nanomolar; conc., concentration; ref., reference.

To determine the efficiency of the PCR, cycle thresholds obtained from a series of 5 template DNA dilutions of at least 3 different samples were graphed on the y-axis versus the log of the dilution on the x-axis. For *bla*_NDM_, a clinical isolate was used because no positive fecal samples were available. The PCR efficiencies were 16S rDNA, 94.0%; *cfxA*, 99.0%; *tetM*, 97.6%; *tetQ*, 95.9%; *ermB*, 95.5%; *aac(6’)-aph(2”)*, 97.0%; *bla*_CTX-M-1+2_, 98.2%; *bla*_CTX-M-9+2–8-9–25_, 96.7%; *bla*_NDM_, 98.4%; *qnrA*, 97.4%; *qnrB*, 101.0%; and *qnrS*, 102.5%.

We determined PCR detection limits for *bla*_CTX-M_, *qnrB*, and *qnrS*. Clinical isolates harboring these genes were suspended in a 0.5 mol/U McFarland solution, then diluted 10-fold in sterile saline solution. Quantification of CFU in the suspensions was achieved by inoculating blood agar plates (Oxoid) and counting the number of colonies after overnight incubation at 37°C. Next, 20 μL of the quantified suspensions was mixed with 180 μL of feces and submitted to DNA extraction as described above. Subsequently, quantitative PCR was performed on extracted DNA to generate standard curves for quantification. For *bla*_CTX-M_, the detection limit was 12–40 CFU/PCR. For *qnrB* and *qnrS*, the detection limit was 1–5 CFU/PCR.

### Statistical Analyses

We calculated differences in relative resistance gene abundances between samples from before and after travel for each traveler by using the ΔΔCt method with a Pfaffl modification to correct for PCR efficiency (ratio: Etarget^ΔCTtarget/Ereference^ΔCTreference) (*24*), which is the standard method to measure the relative change in mRNA expression levels by using real-time PCR. However, in this study, rather than measuring mRNA expression levels, the relative amount of target DNA present was measured by using this method. The 16S rDNA was used as the reference gene.

To better visualize increases and decreases in gene abundances in graphs, we converted abundance ratios to a fold change. To determine the overall abundance change of a resistance gene, ratios were log-transformed. A 2-tailed, 1-sample *t* test was used to test whether the mean log ratio significantly differed from 0.

The number of fecal samples positive for a resistance gene after travel was compared with the positive samples obtained before travel by using the McNemar test for paired samples. Multivariable logistic regression analyses were used to test for the association between age, sex, travel destination and duration, traveler’s diarrhea, and antimicrobial drug use preceding travel (independent variables) and the acquisition of antimicrobial resistance genes (dependent variable). The association between acquisitions of multiple resistance genes was determined by a χ^2^ test. All analyses were performed by using IBM SPSS Statistics version 20 (www-01.ibm.com/support/docview.wss?uid=swg24029274). Results were interpreted as statistically significant when p was <0.05.

## Results

### Study Population

The study comprised 122 travelers (71 women, 51 men) whose median age was 43 years (range 18–72 years). The median length of stay abroad was 21 days (range 5–240 days). Fourteen participants traveled for >60 days; 5 participants traveled for >120 days. Most participants visited 1 country; 22 visited >1 country. Six participants visited >1 of the defined geographic regions (Table 2); 7 participants did not provide information about their destination.

**Table 2:** Characteristics of 122 travelers observed for rates of antimicrobial resistance gene acquisition after international travel, the Netherlands, 2010–2012*

Characteristic	No. (%)
Sex	
M	51 (41.8)
F	71 (58.2)
Clinical finding	
Traveler’s diarrhea	45 (36.9)
Antimicrobial drug use	15 (12.3)
Region visited	
Southeast Asia	28 (23.0)
Indian subcontinent	31 (25.4)
Northern Africa	16 (13.1)
Southern Africa	17 (13.9)
Southern Europe	6 (4.9)
Central America	4 (3.3)
South America	6 (4.9)
Other/multiple	7 (5.7)

*Median age, y (range) of travelers was 42.7 (18–72) and median travel duration, (range) was 21.0 (5-20) months. Countries in respective regions are as follows: Southeast Asia (Indonesia, Philippines, Malaysia, Myanmar, Cambodia, Thailand, Vietnam), Indian subcontinent (India, Nepal, Sri Lanka), northern Africa (Canary Islands, Egypt, Gambia, Ghana, Togo, Morocco, Senegal, Uganda), southern Africa (Namibia, Kenya, Tanzania, Zanzibar, Mauritius, South Africa), Central America (Panama, Costa Rica, Mexico), South America (Argentine, Bolivia, Brazil, Columbia, Peru, Suriname), southern Europe (Croatia, Spain, Turkey), other (Australia, Fiji, New Zealand, Oman).

### Prevalence of Resistance Genes in Fecal Samples

**Figure 1:**
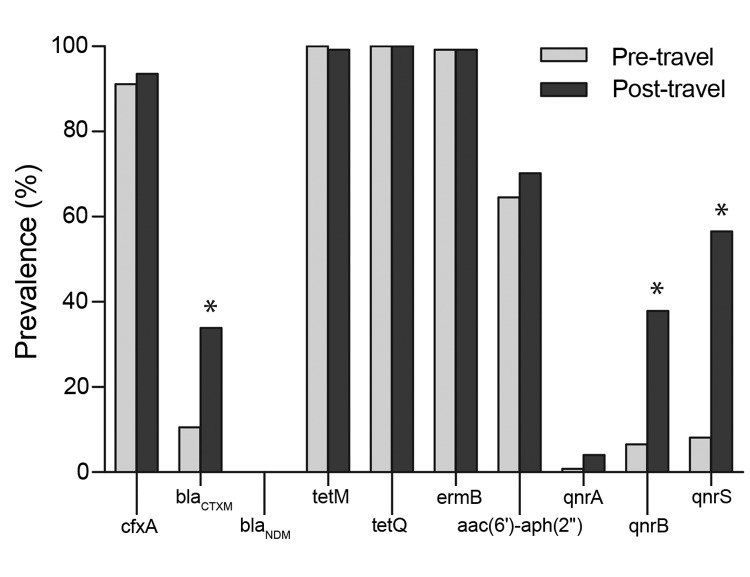
Prevalence (%) of antimicrobial drug resistance determinants in fecal samples from 122 healthy travelers from the Netherlands before and after travel, 2010–2012. Statistical significance of the prevalence between the 2 groups was calculated by using the McNemar test for paired samples and is indicated by * (p<0.001).

Figure 1 shows the prevalence of the antimicrobial drug resistance determinants in fecal samples from the 122 healthy volunteers before and after international travel. The *cfxA* gene was detected in 111 (91.0%) fecal samples before travel and in 115 (94.3%) samples after travel. The ESBL encoding *bla*_CTX-M_ gene was prevalent in 11 (9.0%) before travel and in 41 (33.6%) samples after travel, which was a significant increase (p<0.001). 

After travel, samples from 5 participants contained *bla*_CTX-M_ genes of 2 different phylogenetic groups. Before travel, single CTX-M variant was detected for 2 of these persons, and *bla*_CTX-M_ genes were not detected for the other 3 persons. After travel, the gene was not detected in the samples of 6 persons who were positive for the *bla*_CTX-M_ gene before travel. The carbapenemase-encoding gene *bla*_NDM_ was not detected in any sample.

The prevalence of both *tetM* and *tetQ* was very high in the fecal samples. The *tetM* gene was present in all samples before travel and in 121 (99.2%) samples after travel, and *tetQ* was detected in all samples before and after travel. The prevalence of the *ermB* gene was also high in samples both before and after travel (99.2% for both). The prevalence of the *aac(6′)-aph(2′′);* gene was not altered by traveling; this gene was present in 79 (64.5%) of samples before travel and in 86 (70.5%) samples after travel. 

Before travel, prevalence of the quinolone resistance genes *qnrA*, *qnrB*, and *qnrS* was relatively low: 0.8%, 6.6%, and 8.2%, respectively. After travel, each of the 3 genes increased: *qnrA*, *qnrB*, and *qnrS* were detected in 3.3%, 36.9%, and 55.7% of samples, respectively. *qnrB* and *qnrS* were significantly higher after than before travel (p<0.001).

### Relative Gene Abundance Before and After Travel

**Figure 2:**
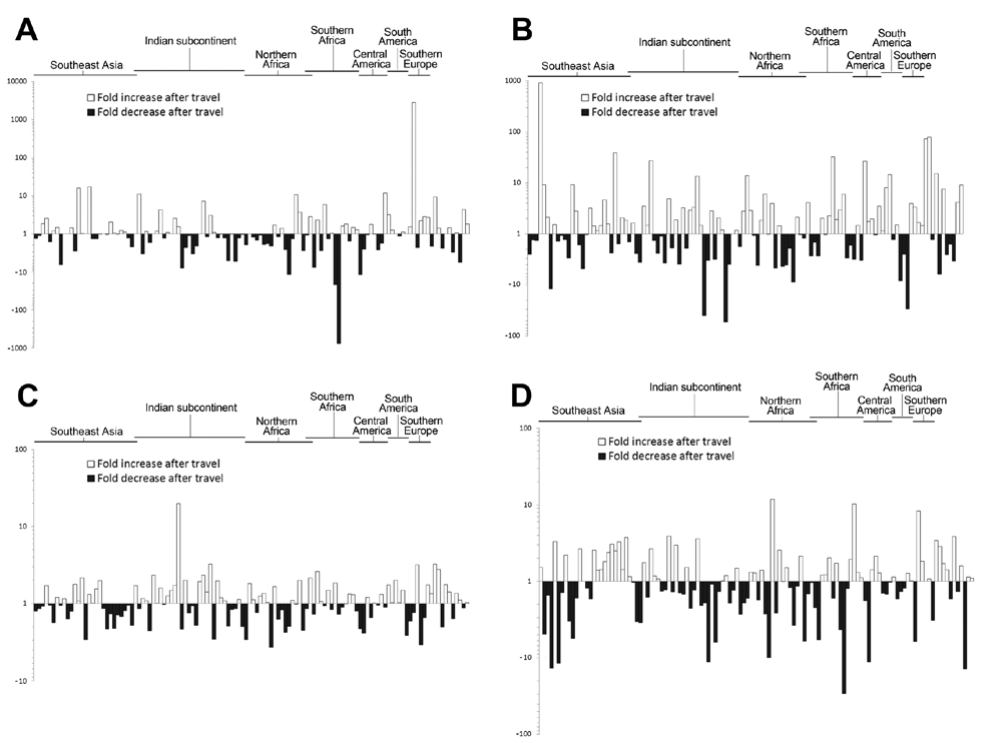
Relative changes in gene abundance before and after travel for each of 122 healthy travelers from the Netherlands during 2010–2012 for genes *cfxA* (A), *tetM* (B), *tetQ* (C), and *ermB* (D). Increases are shown with white bars on the positive *y*-axis; decreases are shown in dark gray bars on the negative *y*-axis. Each bar on the *x*-axis represents the change in a different study participant. The travel destination regions of the participants are indicated above the graph. No region is indicated for some travelers who either visited >1 of these regions or visited countries that were not in the defined regions (see Table 2).

Because the prevalence of the *cfxA*, *tetM*, *tetQ*, and *ermB* genes was very high before and after travel, we compared the relative abundance of the genes in both samples from each traveler to determine whether traveling influenced the gene abundance. For all 4 genes, the observed changes in gene abundance per traveler were distributed between increases and decreases (Figure 2). Determining the overall increase or decrease of the abundance of each gene showed that none of the investigated genes changed significantly (p>0.05 for all) in abundance after travel.

### Effect of Travel Destination and Other Risk Factors on Gene Acquisition

The rate of acquisition of a *bla*_CTX-M_ gene was highest for travelers visiting the Indian subcontinent (58.1%; p<0.05, OR 26.22, 95% CI 2.86–240.38) (Table 3). Travel to other regions was associated with a *bla*_CTX-M_ acquisition rate of 17.9% for Southeast Asia and 31.3% and 29.4% for northern and southern Africa, respectively. In the combined category comprising southern Europe, Central America, and South America, 1 *bla*_CTX-M_ acquisition (6.3%) was detected in a traveler who had been to southern Europe (Turkey).

**Table 3:** Associations between travel-associated risk factors and rates of *bla*_CTX-M_, *qnrB*, and *qnrS* acquisition among 122 healthy travelers from the Netherlands, 2010–2012*

Traveler characteristic	No. travelers	Antimicrobial drug resistance genes acquired by travelers							
		*bla*_CTX-M_			*qnrB*			*qnrS*	
		No. (%)	OR (95% CI)‡		No. (%)	OR (95% CI)‡		No. (%)	OR (95% CI)‡
Region visited									
Europe and America§¶	16†	1 (6.3)	1.00		6 (37.5)	1.00		3 (18.8)	1.00
Southeast Asia	28†	5 (17.9)	3.34 (0.34–33.14)		7 (25.0)	0.47 (0.12–1.90)		**21 (75.0)**	**15.74 (3.13–79.24)**
Indian subcontinent	31†	**18 (58.1)**	**26.22 (2.86–240.18)**		10 (32.3)	0.71 (0.18–2.71)		**19 (61.3)**	**9.23 (1.94–43.87)**
Northern Africa	16†	5 (31.3)	7.28 (0.70–75.92)		5 (31.3)	0.64 (0.14–2.98)		7 (43.8)	2.90 (0.54–15.57)
Southern Africa	17†	5 (29.4)	5.57 (0.56–55.77)		5 (29.4)	0.65 (0.15–2.84)		6 (35.3)	2.41 (0.46–12.66)
Sex									
F§	71	25 (35.2)	1.00		25 (35.2)	1.00		41 (57.7)	1.00
M	51	13 (25.5)	0.62 (0.23–1.67)		15 (29.4)	1.06 (0.44–2.57)		21 (41.2)	0.39 (0.15–1.00)
Antimicrobial drug use									
No§	107	32 (29.9)	1.00		35 (32.7)	1.00		52 (48.6)	1.00
Yes	15	6 (40.0)	1.44 (0.40–5.25)		5 (33.3)	1.28 (0.36–4.51)		10 (66.7)	1.64 (0.43–6.22)
Traveler’s diarrhea									
No§	77	20 (26.0)	1.00		25 (32.5)	1.00		40 (51.9)	1.00
Yes	45	18 (40.0)	1.84 (0.70–4.82)		15 (33.3)	0.97 (0.40–2.37)		22 (48.9)	0.65 (0.26–1.63)
*OR, odds ratio. Boldface indicates statistical significance (p<0.05). †Numbers do not total 122 because the 14 travelers who visited multiple or unknown regions were added to a remainder category not included in this table. ‡ORs and 95% CIs of the associations between risk factor and acquisition of resistance gene (negative before travel and positive after travel) by multivariable logistic regression analysis. Models included the following variables: travel destination, age, travel duration, sex, and antimicrobial drug use within 3 mo. preceding the travel, and traveler’s diarrhea. §Reference category. ¶Southern Europe, Central and South America, previously reported non–high-risk regions, were pooled to establish an adequately sized reference category.									

The acquisition of the *qnrB* gene was not associated with travel to a specific region, whereas the acquisition of *qnrS* was highest for Southeast Asia (75.0%; p = 0.001, OR 15.7, 95% CI 3.1–79.2), and second highest for the Indian subcontinent (61.3%; p<0.05, OR 9.2, 95% CI 1.9–43.9). The acquisition rate was also elevated for northern Africa (43.8%) and southern Africa (35.3%) but not significantly so.

We also investigated associations between age, sex, travel destination and duration, traveler’s diarrhea, and antimicrobial drug use preceding the travel and the acquisition of resistance genes. No associations were found (Table 3).

### Phylogenetic Groups of *bla*_CTX-M_ Genes and Association with *qnr* Genes

Of the 41 *bla*_CTX-M_ genes acquired during travel, 24 belonged to the CTX-M-1 group, 2 belonged to the CTX-M-2 group, 6 were of the CTX-M-9 group, and 9 were positive for the CTX-M-2–8-9–25 probe but not for the CTX-M-2 or 9 probe, indicating that these genes were in groups 8 or 25. The CTX-M groups acquired per region are shown in Table 4. In contrast, 9/11 CTX-M types detected in the pre-travel samples belonged to the CTX-M-9 group and 2/11 to the CTX-M-1 group.

**Table 4:** CTX-M groups of the acquired genes during travels by 122 travelers from the Netherlands, 2010–2012

Region	CTX-M group			
	1	2	9	8/25
Southeast Asia	2	1	3	0
Indian subcontinent	15	0	2	3
Northern Africa	3	0	1	1
Southern Africa	2	0	0	3
Southern Europe	0	1	0	0
Other	2	0	0	2
Total	24	4	6	9

Associations between the acquisitions of the *bla*_CTX-M_ and *qnr* genes were also investigated (Table 5). The acquisition of a *bla*_CTX-M_ gene was not associated with that of a *qnrB* (p = 0.305) or *qnrS* gene (p = 0.080); neither was the gain of a *bla*_CTX-M_ gene of the CTX-M-1 group, which was the dominant acquired type (58.5%) associated with the acquisition of either *qnrB* (p = 0.631) or *qnrS* (p = 0.256).

**Table 5:** Associations between acquisition of *bla*_CTX-M_ and *qnrB* or *qnrS* during travels by 122 travelers from the Netherlands, 2010–2012

*bla*_CTX-M_ acquisition	*qnrB* acquisition, no. (%)			*qnrS* acquisition, no. (%)	
	No	Yes		No	Yes
No	59 (70.2)	25 (29.8)		46 (54.8)	38 (45.2)
Yes	23 (60.5)	15 (39.5)		14 (39.8)	24 (63.2)

## Discussion

We used a metagenomic approach to study effects of international travel on part of the resistome of the human gut microbiota. Our results provide insights into the prevalence of the investigated resistance genes in the human gut microbiota and demonstrate high rates of acquisition of the ESBL encoding gene *bla*_CTX-M_ and quinolone resistance encoding genes *qnrB* and *qnrS* related to international travel. The prevalence of these genes increased from 9.0%, 6.6%, and 8.2% before travel to 33.6%, 36.9%, and 55.7% after travel, respectively.

Prospective cohort studies among travelers from Australia (*13*), the Netherlands (*16*), and Sweden (*14*,*15*) showed that international travel was a risk factor for colonization with ESBL-producing *Enterobacteriaceae* spp. and that travel to India or the Indian subcontinent was the highest risk factor. These findings agree with the rates of *bla*_CTX-M_ acquisition found in our study, which were highest for travelers to the Indian subcontinent.

The phylogenetic types of the *bla*_CTX-M_ gene that were acquired in our study group were clearly dominated by CTX-M group 1, especially in the Indian subcontinent. This geographical association corresponds to the aforementioned cohort studies (*13**–**16*), which showed that ESBL-producing *Enterobacteriaceae* identified in travelers to India or the Indian subcontinent mainly comprise CTX-M group 1. Although the statistical power of our study was insufficient to analyze the specific CTX-M groups, it was striking that genes of the CTX-M-2 group were detected twice and those of either group 8 or 25 were detected 9 times. In previous studies, these CTX-M groups were not detected at all (*13*,*14*) or were detected only sporadically (*15*,*16*). The difference in results could be caused by our use of a metagenomic approach, which might detect *bla*_CTX-M_ in a much wider array of species than did studies investigating specific cultured *Enterobacteriaceae* spp. This difference in approach might furthermore explain that of the *bla*_CTX-M_ genes detected before travel in the population in our study, most (9/11, 82%) were of the CTX-M-9 group, which contrasts studies that report that *bla*_CTX-M-15_ (which belongs to the CTX-M-1 group) is predominant in ESBL-producing *Enterobacteriaceae* in the Netherlands (*16*,*25*,*26*). Aside from the different method used, the population sizes in these studies were larger than the cohort in our current study.

Plasmid-mediated quinolone resistance genes, such as the *qnr* variants, provide low-level quinolone resistance. However, these genes are relevant because they facilitate the emergence of higher-level resistance and thus can speed the development and spread of resistance to these antimicrobial agents (*27*). Although foreign travel has been associated with the acquisition of plasmid-mediated quinolone resistant–positive isolates (*28*–*30*), these genes have thus far not been focused on in prospective cohort studies investigating the effects of travel on antimicrobial resistance.

A study by Vien et al. that investigated the prevalence of the *qnr* genes in fecal swab samples from children in Vietnam who had acute respiratory tract infections (*23*) showed very high *qnrS* prevalence (74.5%). Travel to areas with such a high prevalence could be a major risk factor for acquisition of these genes. Five (83%) of 6 participants in our study who had traveled to Vietnam acquired a *qnrS* gene. In total, 11 volunteers had traveled to Cambodia, Thailand, Vietnam, or a combination of these geographically neighboring countries, and 9 (82%) acquired a *qnrS* gene. These data suggest that organisms carrying the *qnrS* gene are highly prevalent in these areas and that travelers visiting these areas have a high risk for exposure to those organisms.

Coexistence of *qnr* genes with various other resistance genes, such as *bla*_CTX-M_, on the same plasmid is well known (*31*–*34*) and could be related to our finding that both types of genes were more prevalent in the study participants’ samples after travel. However, we found no association between these genes in these samples. The *qnrS* gene was most often acquired by travelers who visited Southeast Asia and, to a lesser extent, the Indian subcontinent, whereas the acquisition rate for *bla*_CTX-M_ was clearly highest for travelers to the Indian subcontinent but was not higher for travelers to Southeast Asia than for travelers to other regions. These findings indicate that although travel to the Indian subcontinent is a high-risk factor for acquiring both of these genes, these risk factors are not necessarily related.

Compared with culturing methods, a metagenomic approach has the advantage of being able to detect resistance in a much wider array of species; however, a limitation is that it is not yet known in which organisms the acquired resistance genes detected in our study are present, nor if they are being expressed. Another limitation of our study is that the study population was not large enough for us to conduct a more extensive risk analysis. Future studies that conduct more extensive analyses for risk factors, such as antimicrobial drug use, travel destination, and duration of travel, would benefit from larger populations. Furthermore, in future studies inclusion of a follow-up sampling of travelers would be highly relevant for investigating the period in which these acquired resistance genes remain in the resistome and if the perseverance or even HGT of these genes in the resistome is promoted by factors such as selective pressure introduced by antimicrobial drug use. Little is known about the duration of travel-acquired resistant organisms in the human microbiota, although their continued viability plays a key role in the ability to further spread these organisms or resistance elements.

During our investigation of several targeted resistance genes, it became evident that resistance genes from foreign environments are being introduced into the gut resistome at high rates related to international travel. Although the consequences of these changes in the resistome are difficult to predict, the introduction of these genes into the genetic pool of resistance elements may create opportunities for the horizontal transfer to other organisms in the gut microbiota.

Our study data demonstrated an increasing prevalence of *bla*_CTX-M_, *qnrB*, and *qnrS* genes in the feces of healthy volunteers from the Netherlands immediately after they returned from international travel. These findings contribute to the increasing evidence that travelers contribute to the spread of antimicrobial drug resistance.
